# Benchmarking a GATE/Geant4 Monte Carlo model for proton beams in magnetic fields

**DOI:** 10.1002/mp.13883

**Published:** 2019-11-13

**Authors:** Fatima Padilla‐Cabal, Jose Alejandro Fragoso, Andreas Franz Resch, Dietmar Georg, Hermann Fuchs

**Affiliations:** ^1^ Department of Radiotherapy Medical University of Vienna/AKH Vienna Austria; ^2^ Christian Doppler Laboratory for Medical Radiation Research for Radiation Oncology Medical University of Vienna Vienna Austria; ^3^ Department of Nuclear Physics Higher Institute of Technologies and Applied Science Havana Cuba

**Keywords:** beam modeling, magnetic fields, MC simulations, proton therapy

## Abstract

**Purpose:**

Magnetic resonance guidance in proton therapy (MRPT) is expected to improve its current performance. The combination of magnetic fields with clinical proton beam lines poses several challenges for dosimetry, treatment planning and dose delivery. Proton beams are deflected by magnetic fields causing considerable changes in beam trajectories and also a retraction of the Bragg peak positions. A proper prediction and compensation of these effects is essential to ensure accurate dose calculations. This work aims to develop and benchmark a Monte Carlo (MC) beam model for dose calculation of MRPT for static magnetic fields up to 1 T.

**Methods:**

Proton beam interactions with magnetic fields were simulated using the GATE/Geant4 toolkit. The transport of charged particle in custom 3D magnetic field maps was implemented for the first time in GATE. Validation experiments were done using a horizontal proton pencil beam scanning system with energies between 62.4 and 252.7 MeV and a large gap dipole magnet (B = 0–1 T), positioned at the isocenter and creating magnetic fields transverse to the beam direction. Dose was measured with Gafchromic EBT3 films within a homogeneous PMMA phantom without and with bone and tissue equivalent material slab inserts. Linear energy transfer (LET) quenching of EBT3 films was corrected using a linear model on dose‐averaged LET method to ensure a realistic dosimetric comparison between simulations and experiments. Planar dose distributions were measured with the films in two different configurations: parallel and transverse to the beam direction using single energy fields and spread‐out Bragg peaks. The MC model was benchmarked against lateral deflections and spot sizes in air of single beams measured with a Lynx PT detector, as well as dose distributions using EBT3 films. Experimental and calculated dose distributions were compared to test the accuracy of the model.

**Results:**

Measured proton beam deflections in air at distances of 465, 665, and 1155 mm behind the isocenter after passing the magnetic field region agreed with MC‐predicted values within 4 mm. Differences between calculated and measured beam full width at half maximum (FWHM) were lower than 2 mm. For the homogeneous phantom, measured and simulated in‐depth dose profiles showed range and average dose differences below 0.2 mm and 1.2%, respectively. Simulated central beam positions and widths differed <1 mm to the measurements with films. For both heterogenous phantoms, differences within 1 mm between measured and simulated central beam positions and widths were obtained, confirming a good agreement of the MC model.

**Conclusions:**

A GATE/Geant4 beam model for protons interacting with magnetic fields up to 1 T was developed and benchmarked to experimental data. For the first time, the GATE/Geant4 model was successfully validated not only for single energy beams, but for SOBP, in homogeneous and heterogeneous phantoms. EBT3 film dosimetry demonstrated to be a powerful dosimetric tool, once the film response function is LET corrected, for measurements in‐line and transverse to the beam direction in magnetic fields. The proposed MC beam model is foreseen to support treatment planning and quality assurance (QA) activities toward MRPT.

## 1 Introduction

After the clinical implementation of magnetic resonance guidance in external beam therapy using photon beams (MRXT), research on MR‐guided ion beam therapy increased in the last years.^1^, ^2^, ^3^, ^4^, ^5^, ^6^, ^7^ First studies on the feasibility of MRPT were already performed from a technical^5^, ^8^ and dosimetric point^1^, ^3^, ^4^ of view, showing that proper compensations for lateral beam bending due to magnetic fields are strictly required. To predict the effect of magnetic fields on beam trajectories and dose calculations, analytical^2^, ^9^, ^10^, ^11^, ^12^, ^13^ and full‐based Monte Carlo (MC) methods^1^, ^3^, ^4^, ^6^, ^14^ can be employed.

The accuracy of dose calculations for a future MRPT system relies on a complete and detailed description of all single pencil beam interactions not only with homogenous, but also with fringe magnetic fields.^9^, ^13^ For each specific MRPT design, it is henceforth required to create an accurate beam model describing the amount of lateral and angular deflection of every single beam path toward and within the patient. An experimental validation of the proposed beam model is then required to ensure that all possible sources of deflection due to different magnetic fields present on the beam line and the MR core have been considered. A method for the experimental verification of simulated beam deflections and Bragg peak retractions due to the influence of external magnetic fields using Geant4 was recently reported.^15^ Planar dose distributions were measured using EBT3 films, placed horizontally in a PMMA phantom, for collimated proton beams passing through a C‐shaped permanent magnet (B = 0.95 T). The accuracy of MC simulations for single proton pencil beam trajectory calculations in homogeneous phantoms as well as the feasibility of EBT3 film measurements to estimate the beam lateral bending and the Bragg peak retraction was demonstrated.^15^ However, the experiments were limited to irradiations using only single beams and a homogeneous PMMA phantom. In addition, dose distributions transverse to the beams were not measured due to the geometry of the experimental setup and no direct comparison was done between simulated and measured doses, because of the limitations of Gafchromic film dosimetry. The energy‐dependent response of Gafchromic EBT3 films makes their use for particle therapy more difficult compared to photon therapy.^16^, ^17^, ^18^, ^19^ For clinical proton beams, the dose obtained from films can be underestimated up to 20% in the Bragg peak region.^16^, ^20^, ^21^ To correct for the LET quenching, several models are available in literature.^22^, ^23^, ^24^, ^25^, ^26^ For treatment planning or commissioning procedures for a future MRPT system, a complex experimental validation is required. EBT3 films are suitable detectors to account for parallel and transverse 2D dose distributions; however, LET corrections are required under typical proton clinical treatment conditions.

MC simulations are frequently employed in ion beam therapy to support several aspects of beam delivery system, treatment planning and QA especially during the design and commissioning phase of new facilities.^27^, ^28^, ^29^ For a future MRPT system, the development and benchmarking of specific MC beam models, considering proton beam interactions with resultant magnetic fields, are essential to provide reliable input data for dose calculations and to reduce the amount of measuring times considerably. The GATE/Geant4 package was demonstrated to be a powerful tool for beam modeling and dose calculation in ion beam therapy.^30^, ^31^, ^32^, ^33^ Likewise, Geant4 demonstrated high accuracy describing and propagating particle tracks in a variety of electromagnetic fields, regardless their homogeneity.^34^

This work aims to develop and validate a GATE/Geant4 MC beam model for clinically relevant proton beams interacting with external heterogeneous magnetic fields up to 1 T. The performance of the model describing the beam optics and the energy spectrum were evaluated using in‐depth and lateral dose profiles. For this purpose, planar dose distributions were determined using EBT3 films placed both parallel and transverse to the beam. The beam model was validated including not only single pencil beams, but also spread‐out Bragg peaks (SOBP) and irradiations were done in homogeneous and heterogeneous phantoms. Finally, film measurements were corrected by LET quenching using a recently proposed model, allowing a more reliable comparison between our dosimetric calculations and measurements. The model is foreseen to be used for the generation of input calibration data for treatment planning systems (TPS) and to support dose calculations and QA procedures for a possible future MRPT unit.

## 2 Materials and Methods

### 2.1 Monte Carlo simulations

Monte Carlo simulations were conducted using an extended version of the GATE8.0/Geant4.10.03.p03 toolkit, encompassing a complete in‐house established proton beam line model,^35^ the treatment nozzle, and a 3D field map of a research dipole magnet. The physics models and simulation parameters were selected based on the previous experience from our research group and recommendations from the GATE/Geant4 collaboration.^31^, ^32^, ^33^, ^36^, ^37^ All simulations were performed using the ROOT^38^ framework in version 6.10, Geant4^34^ release 10.3 patch 03, and GATE^31^ version 8.0. Electromagnetic and hadronic processes were described using the QBBC_EMZ physic list, where nuclear interactions are described using QBBC and multiple Coulomb scattering processes using the WentzelIV^39^ model (option 4, EMZ).^33^, ^37^, ^40^ Material compositions and ionization potential were obtained from the NIST‐PSTAR databases.^41^ The corresponding simulation settings used through this work are presented in Table 1.

**Table 1:** GATE simulation settings used through all our calculations.

Simulation settings		
Ionization potential (eV)	Air	85.7
	PMMA	71.0
	Bone (PTFE)	99.1
Density (g/cm^3^)	Air	1.29e‐3
	PMMA	1.19
	Bone (PTFE)	2.13
Production cuts (γ, e−, e+) (mm)	World	10
	Nozzle	1
	Phantom	0.1
Maximum step size (mm)	World	10
	Nozzle	10
	Phantom	0.1
Physic list	QBBC_EMZ	

The MedAustron proton pencil beam scanning (PBS) delivery system was simulated in GATE using the *TPSPencil‐Beam* source and a dedicated beam model including all nozzle elements.^28^, ^35^ Individual pencil beam physical properties at the nozzle exit (energy, position, beam spot size, and divergence) were calculated using a set of polynomial equations obtained from depth–dose profiles and spot reference measurements in the dedicated research room of the MedAustron (EBG MedAustron GmbH, Wiener Neustadt, Austria) ion beam therapy center.^35^, ^42^

#### 2.1.1 Implementation of magnetic field maps in GATE

Beam interactions with magnetic field maps were implemented in GATE 8.0 in similar way to the steps described in the Geant4 *Purging Magnet* example.^43^ A new class *GateMagTabulatedField3D* was introduced to generate a magnetic vector field map with three (**Bx,By,Bz**) components from a custom look‐up table (LUT). This vector field map was used to propagate the particle tracks inside the magnetic field. To integrate the equation of motion of the charged particles in magnetic fields, different numerical methods, defined as steppers, are implemented in Geant4.^44^ Once a method is chosen, particle tracks in the magnetic field are calculated breaking the curved path into linear chord segments.^44^ Several steppers and chord reconstruction parameters from the Geant4 classes *G4MagIntegratorStepper* and *G4ChordFinder* were made accessible within the GATE implementation to increase the accuracy of particle track's propagation. To evaluate the simulation performance in terms of accuracy and calculation times, different stepper orders were initially tested in a pilot phase of the project. The deflection of proton beams (148.2 and 198 MeV) within a custom 3D magnetic field in air was estimated for different steppers and chord reconstruction parameters. The calculated trajectories showed differences on the predicted lateral beam bending always lower than 0.1 mm, the *ClassicalRK4* method being the fastest. Finally, all chord reconstruction parameters were simultaneously decreased, and trajectories were estimated using the *ClassicalRK4* stepper*.* Similar identical results for trajectory reconstruction (<0.1 mm) were found using the maximum and minimum values of the chord reconstruction parameters, while calculation time increased up to a factor of 2.4. From the results of this pilot test, displayed in Table 2, the Geant4 recommended values and a fourth‐order Runge–Kutta stepper were used within all our calculations.

**Table 2:** Default chord reconstruction parameters and steppers values from *G4ChordFinder and G4FieldManager* implemented in the *setMagTabulateField3D* function. The ratio between the calculation times using the lowest tested values in GATE over the recommended Geant4 values is presented.

Parameter	Recommended value Geant4	Tested values GATE	Calculation times ratio
setIntegratorStepper	ClassicalRK4	ClassicalRK4	1
		Simple RK	5.3
		Implicit Euler	5.4
		Explicit Euler	12.9
		Simple Heum	2.0
setStepMinimum	0.01 mm	1 μm–0.01 mm	2.4
setMissDistance	0.25 mm	1 μm–0.25 mm	
setDeltaIntersection	<0.001 mm	1 nm–0.001 mm	
setDeltaOneStep	<0.01 mm	1 nm–0.01 mm	
setMinimumEpsilonStep	5e‐5	1e‐10‐5e‐5	
setMaximumEpsilonStep	0.001	1e‐11‐0.001	

### 2.2 Experimental setup

**Figure 1:**
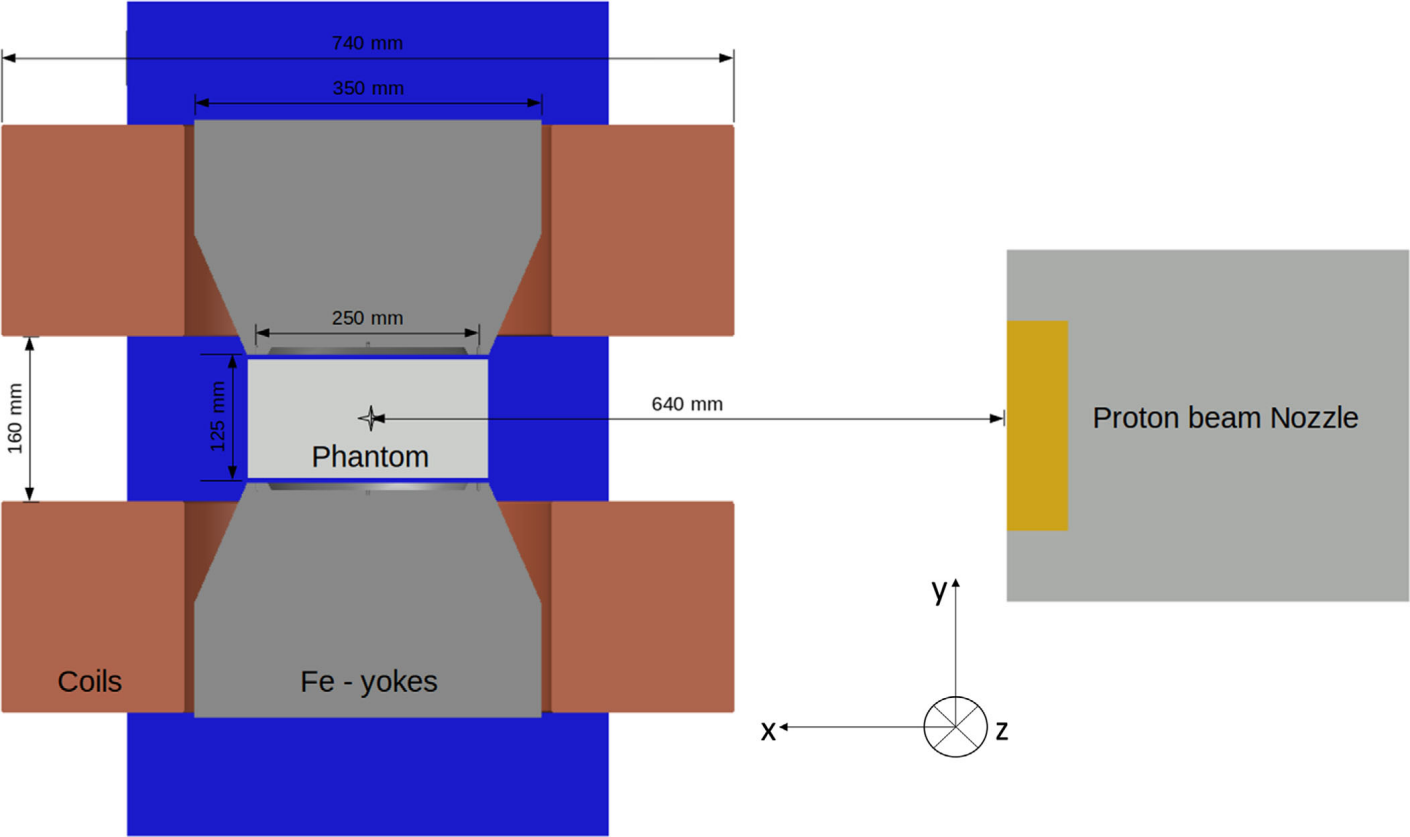
Sketch of the proton beam line model used for the Monte Carlo simulations and experimental measurements. Beam direction is set in the x axes and the geometric center of the magnet is placed at the isocenter, 64 cm downstream the Nozzle exit. [Color figure can be viewed at http://www.wileyonlinelibrary.com]

Measurements were conducted in the horizontal PBS proton beam line in the dedicated research room at the MedAustron ion beam therapy center,^42^ see Fig. 1. Proton beam energies in the range between 62.4 and 252.7 MeV, corresponding to ranges in water from 30 to 350 mm, were employed.

A dipole research magnet (Danfysik A/S, Taastrup, Denmark) was placed in the room isocenter, generating a static magnetic field (B = 0–1 T) perpendicular to the beam incidence direction. The homogeneous field region (magnetic field inhomogeneities below 0.3%) was located in a sphere‐shaped volume of 75 mm radius from the center of the magnet, according to technical specifications. The constrained space between the magnet poles (135 mm) and the magnetic field homogeneity region limited the test geometries and experimental procedures.

**Figure 2:**
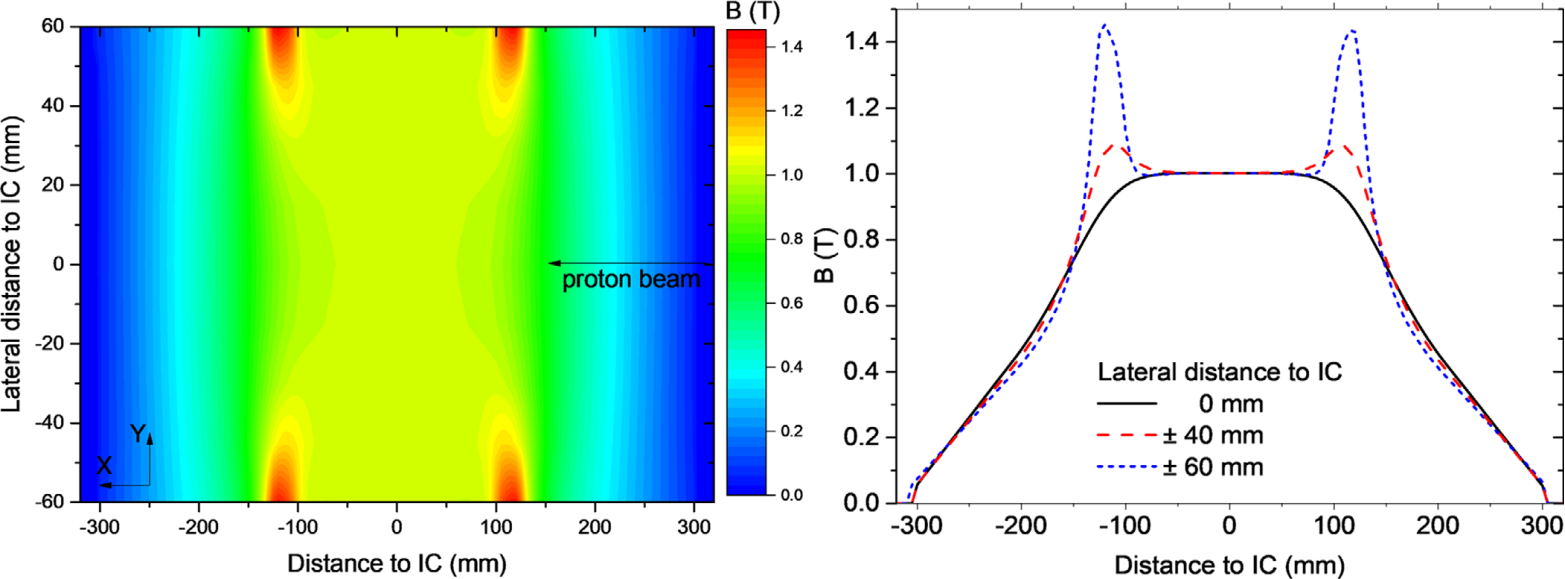
Bidimensional magnetic field map measured at the central plane (Z = 0) of the research magnet (left) and the corresponding linear profiles at three lateral distances from the isocenter (right). [Color figure can be viewed at http://www.wileyonlinelibrary.com]

Measured magnetic field data provided by the manufacturer, including the homogeneous and parts of the fall‐off region, were used to create a vector field map LUT. To reconstruct the complete fringe fields, a 3D linear extrapolation was used to extend the manufacturer field map to the volume where magnetic field intensities were higher than 50 mT. Extrapolated results were compared with measured data of an extended profile from the field map in the beam direction, showing good agreement also in the fall‐off region down to 50 mT. Through this work, vector field LUT was generated in a cartesian volume of dimensions X,Y,Z = 700, 420, 700 mm^3^ using a grid spacing of ΔX = 5 mm and ΔY = ΔZ = 15 mm. Magnetic field intensities were measured at several reference points using an AS‐NTM Transverse Probe coupled to a FM 302 Teslameter (Projekt Elektronik Mess‐ und Regelungstechnik GmbH, Germany), showing a close agreement (below 1%) with the data reported by the manufacturer. A detailed view of the magnetic field maps is shown in Fig. 2.

**Figure 3:**
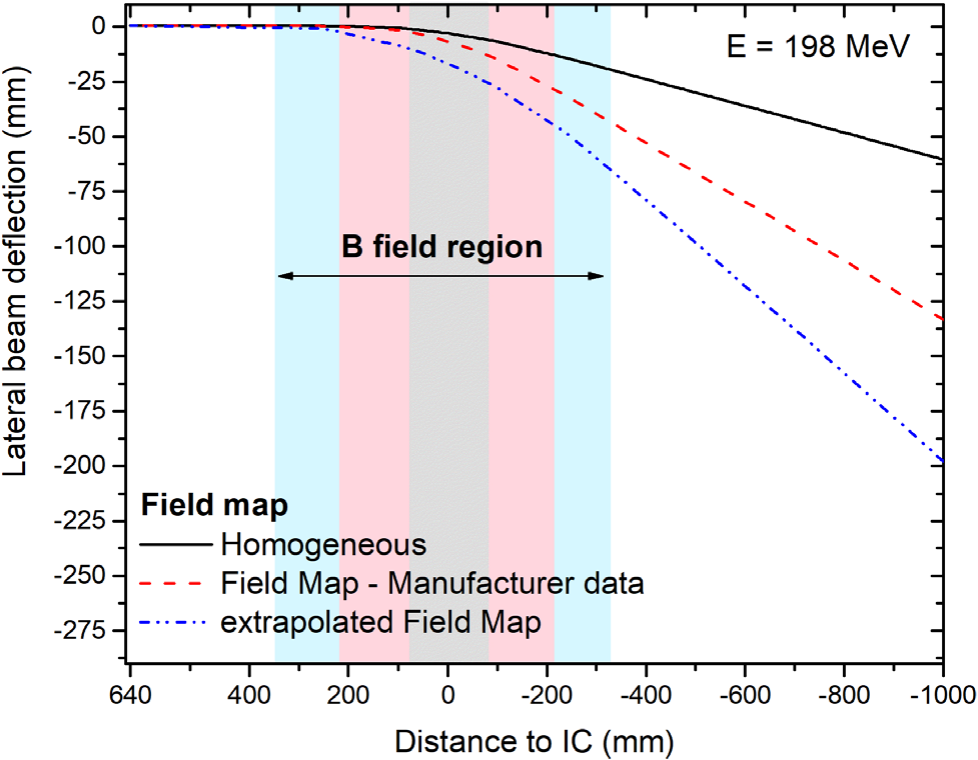
Simulated lateral beam deflection of proton beams accounting for all components of the magnetic field map. The three field map regions are highlighted in the graph in light gray (homogeneous region only), pink (manufacturer measured data), and cyan (extrapolated data), see online color version. [Color figure can be viewed at http://www.wileyonlinelibrary.com]

Trajectories of a 198 MeV proton beam considering the different components of the magnetic field maps were simulated to assess the influence of each magnetic field region on the lateral beam deflection, see Fig. 3. The proton beams originated from the nozzle exit, corresponding to a distance of 640 mm from the isocenter. Considering that the incomplete fringe fields description resulted in a remarkable underestimation on beam bending, the extrapolated field map was used in all our simulations.

### 2.3 Validation procedure

#### 2.3.1 Proton trajectories in air

Lateral beam deflections, scored at different distances from the isocenter, were used to predict beam trajectories in air for single pencil beams (E = 97.4, 148.2, 198 and 252.7 MeV) interacting with magnetic field strengths of B = 0, 0.5 and 1 T. Measured and simulated lateral coordinates were compared to assess the accuracy of the MC model.

Proton beam spots were acquired using a Lynx detector (IBA Dosimetry, XYZ, Germany), consisting of a fluorescent screen, a CCD camera and a mirror. 600 × 600 pixel images with a pixel side length of 0.5 mm were recorded at isoscenter to detector surface distances (ISD) of 465, 665, and 1155 mm, after passing through the research magnet. Each spot was analyzed using the Lynx QA software^45^ (EBG, MedAustron, Wr. Neustadt, Austria), fitting Gaussian distributions on the vertical and horizontal line profiles. The mean and the FWHM were extracted to quantify the beam positions and widths. Lateral deflections were obtained from the difference of the beam positions measured with and without applied magnetic field.

Proton beam lateral deflections and spot widths in air at several distances from the isocenter were simulated in GATE using 15 phase space actors, oriented transverse to the beam direction. Transverse profiles were extracted and fitted to Gaussian distributions using the commercial software MATLAB R2016b, (MathWorks, Inc., Natick, Massachusetts, USA). The mean and FWHM values were validated against the experimental values. The number of protons per simulation was set to 10^6^ to ensure a statistical variance below 1% for the number of particles entering the phase space volume in our beam spot calculations.

#### 2.3.2 Dose distributions

Dose distributions of clinical proton beams in magnetic fields were estimated using the developed MC model and compared to EBT3 films measurements. Gafchromic EBT3 film response showed a negligible influence of magnetic fields up to 1 T,^46^ consequently were used for all the validation measurements.

**Figure 4:**
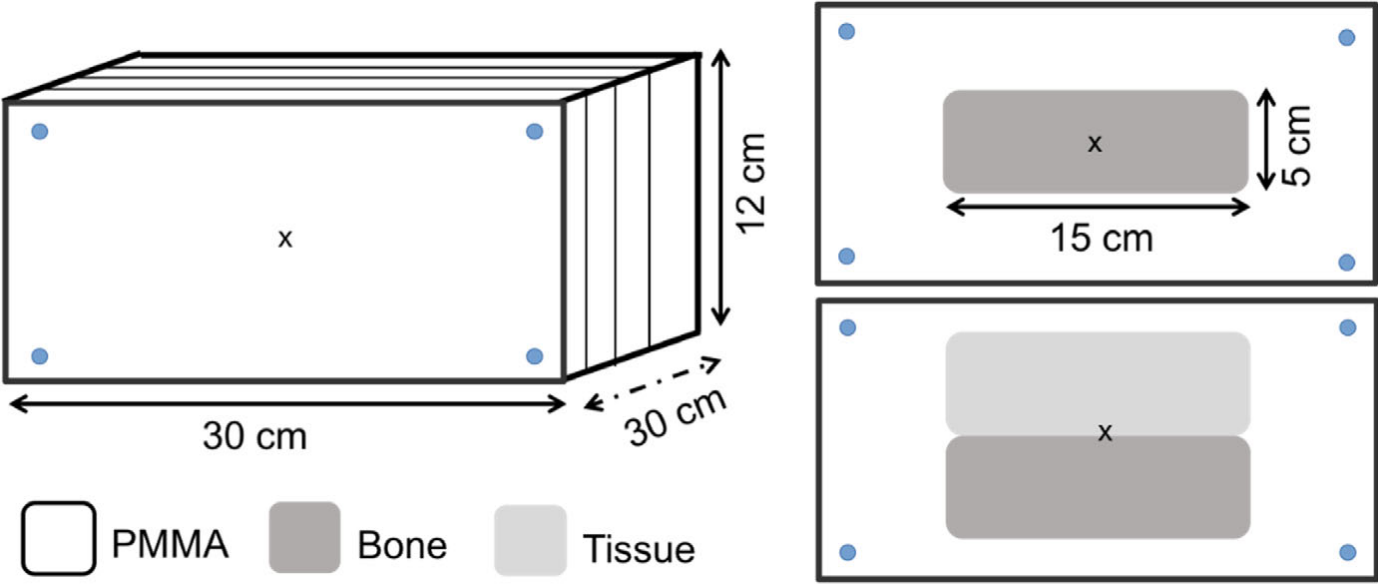
Homogeneous and heterogeneous phantom setups used for the Monte Carlo model benchmarking. [Color figure can be viewed at http://www.wileyonlinelibrary.com]

Dosimetric measurements were conducted using an in‐house built PMMA slab phantom with dimensions of 200 × 120 × 300 mm^3^, located in the center of the magnet. Planar dose distributions were measured using the films in two different configurations: parallel and transverse to the beam. For each irradiation, films were placed in a stack of two or three pieces each and the results were averaged. Reference marks were placed on the film edges to correct and re‐align the measured planar doses positions using translation and/or rotation during post‐processing. During all our measurements, films were placed between PMMA slabs, potentially creating an air gap between the films and continuous slabs. A fixation system based on plastic screws was placed at the phantom corners pressing the slabs together to minimize the dose enhancement ratio effect^47^ in the air‐material interface. Further details about film handling and analysis can be obtained from previous results of our research group.^18^, ^19^, ^46^ Besides the measurements with the homogeneous PMMA phantom, two heterogenous material configurations were investigated. Material (bone and tissue) slabs of 20 × 50 × 150 mm^3^ were placed at the phantom entrance in two geometrical configurations: centrally and creating a lateral interface, see Fig. 4.

Irradiations were performed using two beam modalities—single energy fields and spread‐out Bragg peaks. Treatment plans were created using the treatment planning system (TPS) RayStation v5.99 (RaySearch Laboratories, Stockholm, Sweden) and recalculated with the proposed MC model. Single pencil beams (E = 97.4, 148.2 and 198 MeV), 20 × 20 mm^2^ square fields (E = 148.2 MeV) and two box targets 40 × 40 × 40 mm^3^ centered at depths of 75 and 115 mm from the phantom surface were measured, see Table 3. In order to assess the effect of magnetic fields on dose distributions, identical irradiations were always performed with and without magnetic field.

**Table 3:** Setups for the EBT3 dosimetric measurements performed to evaluate the MC model.

Field	Energy (MeV)	Phantom	Film orientation
Single pencil beam	97.4	PMMA	Parallel
	148.2		
	198.0		
Target 40 × 40 × 40 mm^3^ centered at 75 mm depth	91.2–126.5	PMMA	Parallel
Centered at 115 mm depth	124.0–154.2		
Square field 20 × 20 mm^2^	148.2	PMMA	Transverse
		PMMA with central Bone Slab	
		PMMA with lateral interface	

The experimental setups for the abovementioned dosimetric measurements, see Table 3, were exactly reproduced for MC simulations. Dose distributions were scored in cubic voxels of 2 × 2 × 2 mm^3^ covering the full phantom volume. Bidimensional arrays, parallel and transverse to the beam incidence, mimicking the films, were calculated correspondingly for each irradiation setup.

Laterally integrated dose distributions as a function of depth (IDD) and lateral beam profiles at several penetration depths, covering both the plateau and Bragg peak regions, were determined from 2D planar dose distributions. To assess the accuracy of the proposed model, the range R_80_ (the position of the distal 80% dose point in the Bragg peak) between simulated and measured IDD was calculated. Dose differences between experimental and simulated IDDs were estimated for all penetration depths smaller than a reference R_20_ (the position of the distal 20% dose point in the Bragg peak). The average value of the differences (ΔD_mean_), as well as the maximum (ΔD_max_) dose deviations, was also reported as assessment parameter of the performance of the MC beam model. The position of the maximum (μ) as well as the full width at tenth maximum (FWTM) of simulated and experimental lateral beam profiles were determined and the mean value of their differences over all penetration depths (Δμ_mean_, ΔFWTM _mean_) were also used as validation parameters. Calculated and measured IDD and lateral profiles were always normalized to the dose plateau region, at a penetration depth in PMMA of 20% of the R_80_.

#### 2.3.3 LET quenching correction

A beam quality correction factor $g_{Q,Q_{0}}$ was applied to correct the apparent dose $D_{\mathrm{apparent}}$ from film measurements applying the calibration obtained at the reference beam quality *Q_0_*, that is, at 2 cm WET in a 179 MeV proton beam.^25^ According to literature,^48^ it can be derived from the dose‐averaged LET:

(1) $$g_{Q,Q_{0}}^{-1}\left(LET_{d}\right)=1.02-0.0251\mu m/keV\cdot LET_{d}.$$

The dose‐averaged LET was extracted from the LET‐Actor in GATE/Geant4 using the default scoring routine and the LET to water option,^49^ which uses “method C” form Cortes‐Giraldo and Carabe and is, hence, independent of step limiting events.^50^

## 3 Results

### 3.1 Proton beam trajectories

**Figure 5:**
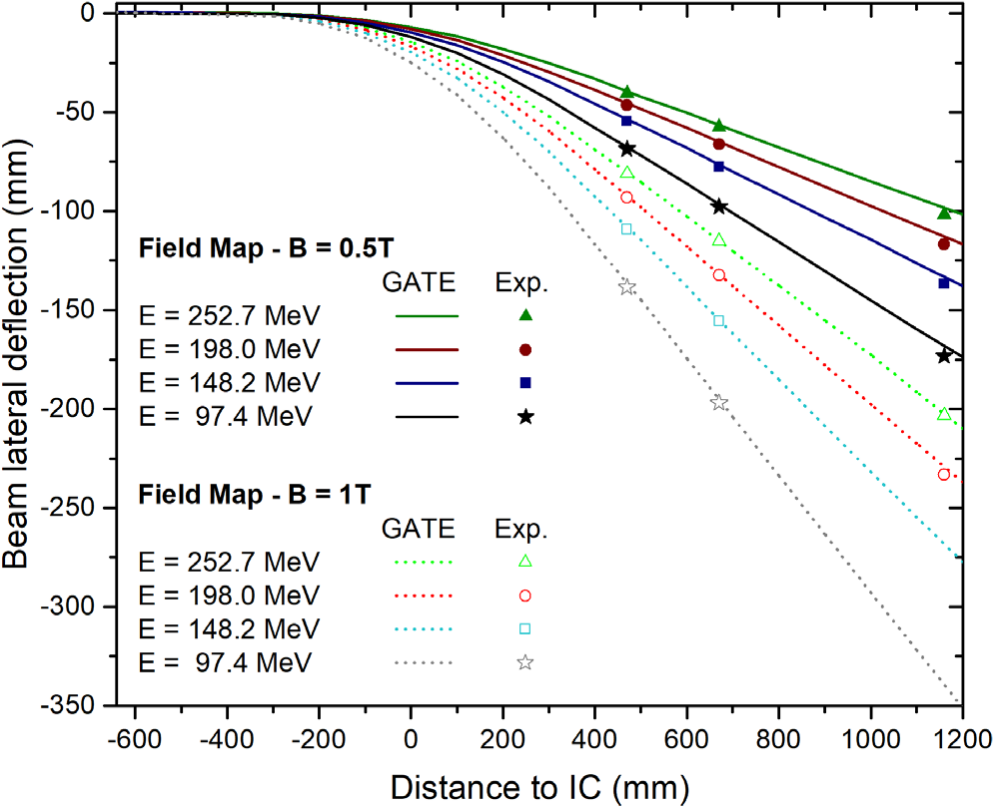
Simulated and measured lateral deflections of proton beams interacting with magnetic field strengths of 0.5 and 1 T. Measurements are displayed as points, while simulated beam trajectories are presented in continuous and dotted lines. [Color figure can be viewed at http://www.wileyonlinelibrary.com]

The comparison between calculated and measured lateral beam deflections in air for four beam energies and two magnetic field strengths is presented in Fig. 5. Differences between the experimental and simulated beam positions were smaller than 2 mm for the two closest distances to the isocenter (46.5 and 66.5 cm), while deviations up to 4 mm were obtained for the farthest distance (115.5 cm). Calculated and measured FWHM values agreed generally within 2 mm. Lateral deflections of the 97.4 and 148.2 MeV at the farthest measurement distance exceeded the Lynx detector dimensions and consequently were not measured.

### 3.2 Dosimetric measurements

#### 3.2.1 Parallel film irradiations

**Figure 6:**
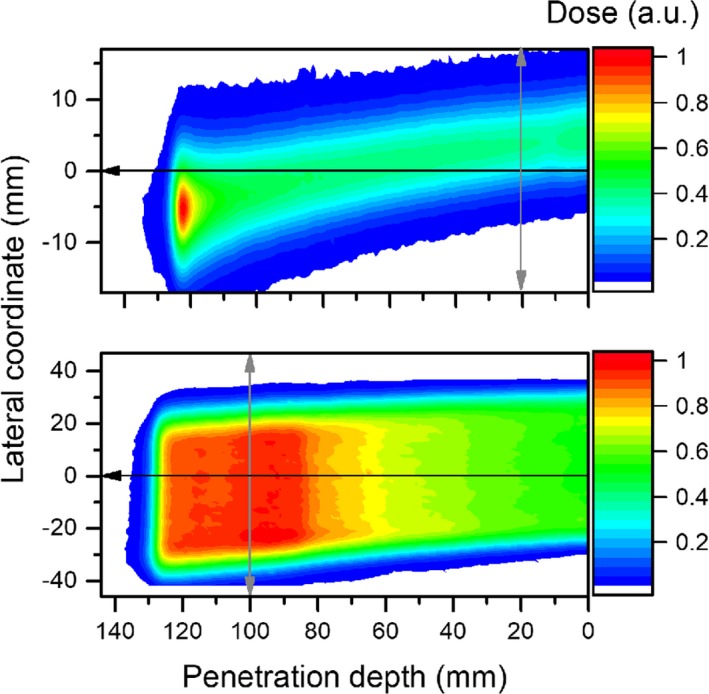
EBT3 measured dose from deflected proton beams passing through a magnetic field (B = 1 T), using a single pencil beam (top) and a 40 × 40 × 40 mm^3^ cubic target (bottom) irradiations. The black and gray arrows indicate the directions in which in‐depth and lateral dose profiles were scored, respectively. [Color figure can be viewed at http://www.wileyonlinelibrary.com]

Planar dose distributions of a 148.2 MeV single pencil beam and a 40 × 40 × 40 cm^3^ SOBP centered at a depth of 115 mm within a B = 1 T magnetic field are shown in Fig. 6. Results are depicted normalized to the maximum dose.

**Figure 7:**
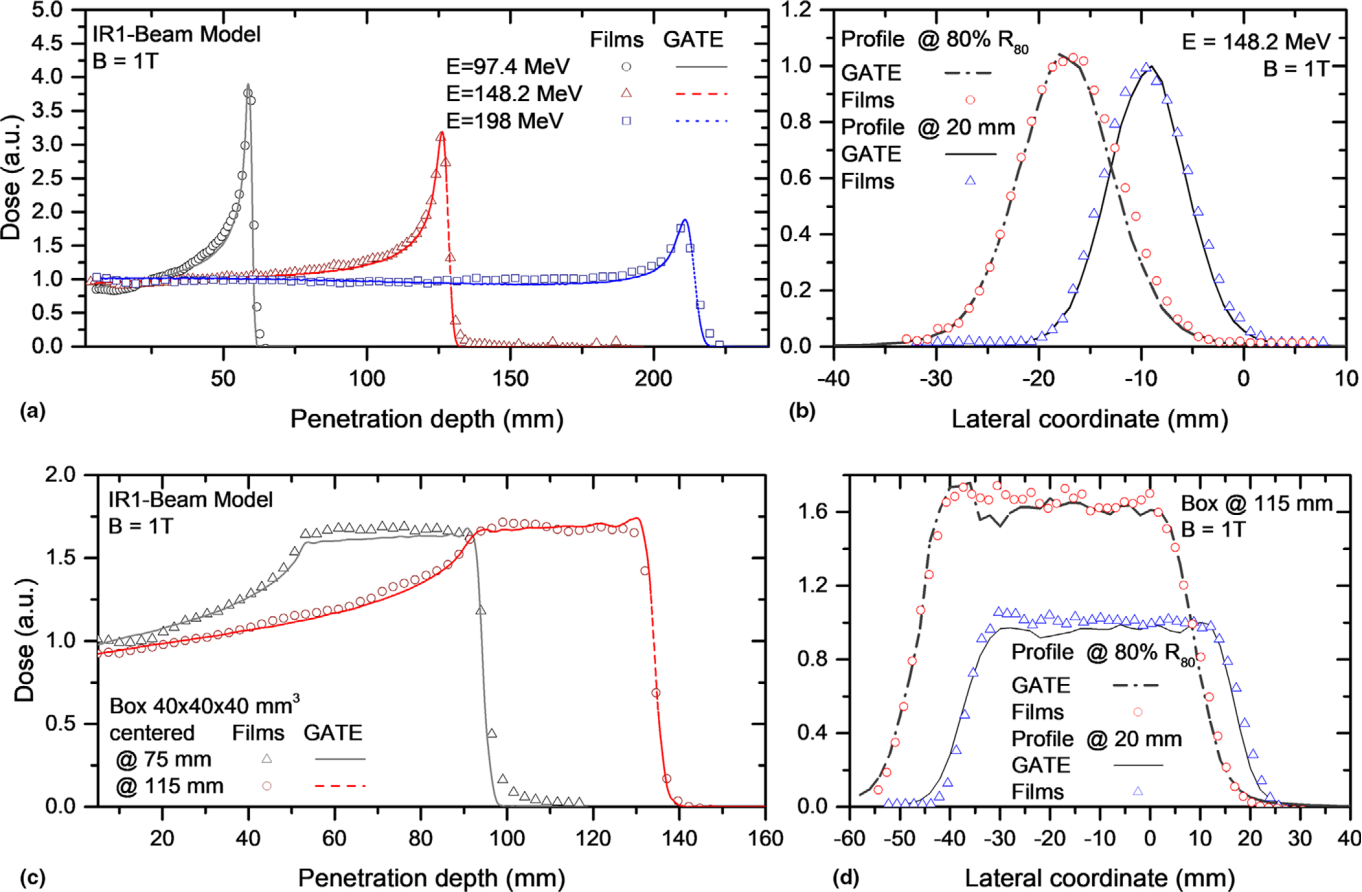
Integrated dose distributions (a,c) and lateral (b,d) profiles measured with EBT3 films and simulated with GATE for proton beams interacting with B = 1 T. The presented experimental values were linear energy transfer corrected. [Color figure can be viewed at http://www.wileyonlinelibrary.com]

Measured and simulated IDDs and lateral dose profiles, for both test settings, are presented in Figs. 7(a)–7(d). A quenching effect in the film response up to 15% was observed and corrected due to the increase in the LET of the protons toward the fall‐off region, with and without magnetic fields. Differences between our MC calculations and experimental data are summarized in Table 4, showing a good agreement of the model.

**Table 4:** Differences between MC and experimental dosimetric parameters for EBT3 films irradiations parallel to the proton beam for magnetic fields strengths of B = 0 and 1 T.

Beam	B (T)	ΔR_80_ (mm)	ΔD_mean/_ ΔD_max_ (%)	Δμ_mean_ (mm)	ΔFWTM_mea0_ (mm)
Single beams					
E = 97.4 MeV	0	−0.1	0.5/6.4	0.2	0.8
	1	0.1	−0.2/7.7	−0.4	−0.7
E = 148.2 MeV	0	−0.1	−0.1/5.6	−0.3	0.8
	1	−0.1	−0.9/5.9	−0.7	0.7
E = 198 MeV	0	0.1	−0.6/4.9	−0.1	−0.3
	1	−0.1	−0.5/8.2	−0.9	−0.4
SOBP 40 × 40 × 40 mm^3^					
Centered at 75 mm	0	−0.1	0.3/5.9	−0.1	−0.1
	1	−0.1	−0.5/7.3	0.4	0.2
Centered at 115 mm	0	0.1	0.1/7.2	0.4	−0.8
	1	0.1	−0.5/8.7	0.8	−1.0

#### 3.2.2 Transverse film irradiations

**Figure 8:**
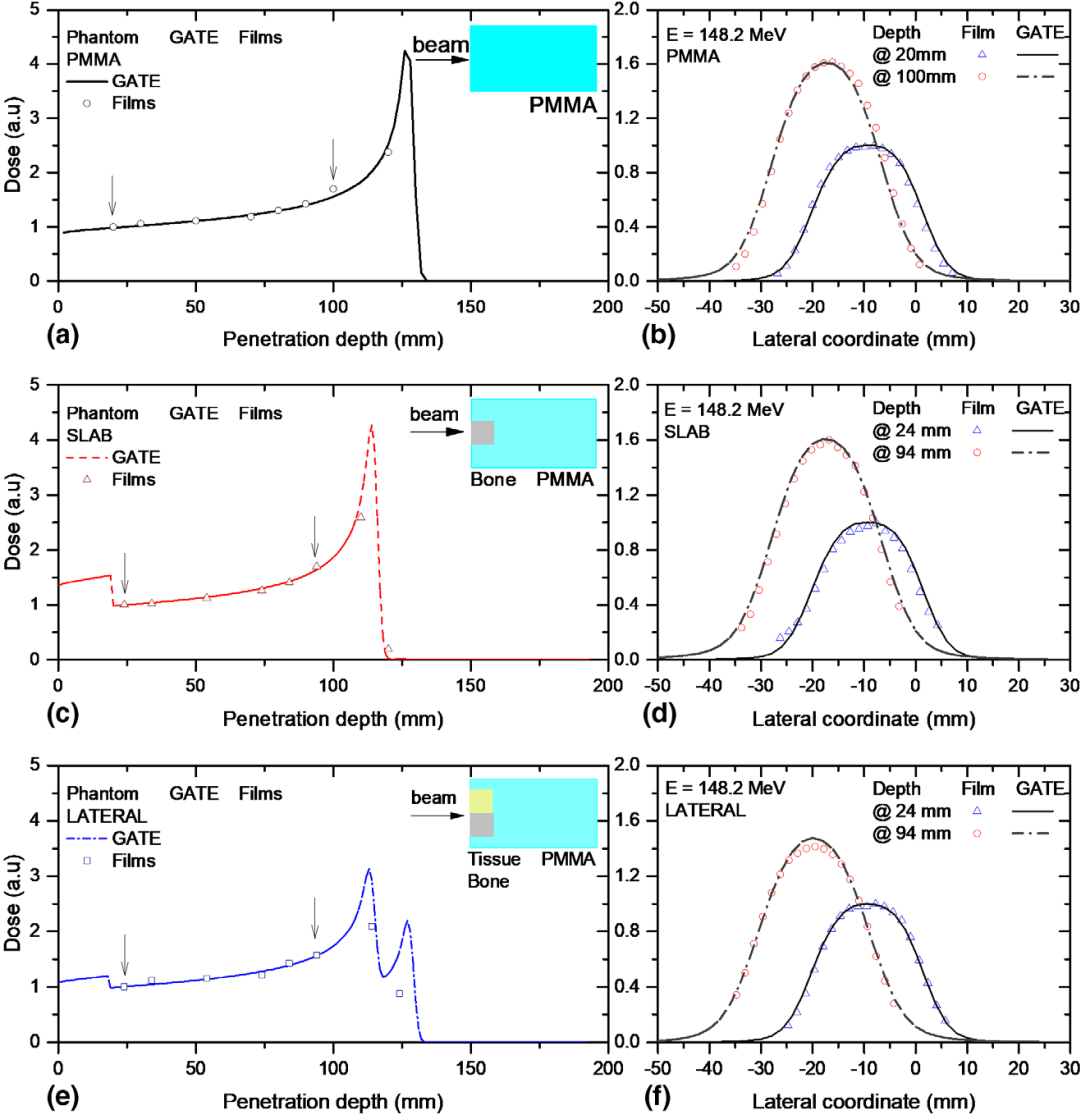
Integrated dose distributions (a,c,e) and lateral (b,d,f) profiles measured with EBT3 films and simulated with GATE for proton beams passing through a magnetic field region of B = 1 T and three phantom material configurations. A simple schematic illustration of the phantom material/geometry is shown as reference on the right top area of the plots. The arrows indicate the positions at which the lateral profiles were scored for comparison. [Color figure can be viewed at http://www.wileyonlinelibrary.com]

IDDs and lateral dose distributions for square field irradiations with a single energy layer (148.2 MeV) are shown in Fig. 8. Results were compared for a proton beam passing through the homogenous PMMA phantom and the two heterogeneous phantom configurations. Lateral beam profiles agreed well with film measurements. Differences between measured and simulated central beam positions and widths were always <1 mm. A retraction of the Bragg peak of 11 mm was observed for the central slab case, while a split Bragg peak was obtained for the lateral interface, see Fig. 8. The shape of the IDD was reproduced with the film measurements for the inhomogeneous phantoms. A higher quenching effect (up to 24 %) was detected in the Bragg peak region and corrected accordingly.

In contrast, the effect of the heterogeneities on lateral profiles was found to be negligible. Neither the lateral beam deflection, nor the beam broadening was affected by the heterogeneities for the two analyzed profiles, scored before the Bragg peak region.

## 4 Discussion

Particle transport using custom magnetic 3D field maps was implemented in GATE for the first time, as far as to our knowledge. In this work, the influence of the path reconstruction parameters and steppers on simulated proton beam trajectories in air was found to be negligible, resulting only in different calculation times. Nevertheless, a more detailed analysis of the influence the chord reconstruction parameters for particles trajectories passing through more complex geometries and heterogeneous material composition may be necessary for future applications.

While describing proton beam trajectories in magnetic fields, it was essential to consider fringe fields as close as possible to reality. In this work, good agreement between the measured and calculated beam deflections (<1.5 mm) could be demonstrated at distances up to 70.0 cm downstream the magnet isocenter, reflecting the accuracy of the employed magnetic field map description and the models employed in the MC simulations. However, higher discrepancies observed at 115.5 cm distance from the isocenter suggest that the interpolation method used for the complete fringe description (intensities higher than 50 mT) might be not fully adequate, or that undetected rotational or positioning errors of the Lynx detector may have occurred. Setup and alignment errors (>1 mm), as well as inaccuracies on the delivered beam spot position (<0.5 mm) from the PBS delivery system can also contribute to the observed discrepancies. The reproducibility on the magnetic field strength over time, accounting for the power supply temporal stability (0.1%) and the accuracy of the field intensity strength using the Hall Probe (0.5%) and the remanence (50 mT) within the magnet, introduced additional uncertainties to the determination of the beam lateral position.

A more detailed evaluation could potentially be performed using a finite element model^9^ to describe also the field components due to beam line scanning magnets or including time‐varying magnetic fields.^13^ In the research beam line used in this work, scanning magnets from the PBS delivery system are located 670.0 and 740.0 cm away from the treatment isocenter. Therefore, their influence on the resultant magnetic field was neglected. The MC simulation method presented in this work could be extended to magnetic fields from typical MRI cores by modifying the 3D field map accordingly.

In this work, film dosimetry was employed considering that noninfluence of the magnetic field on EBT3 film response was observed earlier.^46^ However, typical uncertainties associated with these measurements were higher than the ones expected from measurements using conventional ionization chambers employed for IDD and lateral profiles measurements in proton beam therapy. For example, film reproducibility is well‐known to be limited. Repeatability of EBT3 film dose measurements in the order of 5% were observed in our research group, in contrast to reference measurements using ionization chambers. In addition, an in‐house built PMMA phantom was employed instead of dedicated water phantoms for measurements within the dipole gap. While relative alignment was good, absolute positioning of films shows higher uncertainties. Besides the shortcomings for the phantom alignment within the magnet (~1 mm), the accuracy of the alignment of the films within the phantom (~1 mm) is worse than the absolute detector positioning accuracy used in clinical practice (~0.5 mm or less). Film positioning reproducibility as well as tilting and misalignments during irradiations and scanning were the main sources of uncertainties of our experiments. For example, for parallel film irradiations, misalignments up to 2 mm as well as tilting on angles <0.8° were encountered and corrected during post‐processing. Another factor affecting the dosimetric accuracy is the appearance of lateral response artifacts due to film positioning in the scanner.^51^, ^52^ It is also well‐known, that the local response uniformity of EBT3 films decreases with the dimensions of the analyzed region of interest (ROI),^19^ impairing the quality of dosimetric measurements using large films. Figure 7(c) shows a typical example of inhomogeneous dose distributions on a film measurement. A wobbling effect on the entrance region was observed for the SOBP measured for the shallower target. Although the proposed experimental verification was sufficient for the purpose of this work, for absolute dosimetry, the use of films in parallel orientation is usually not employed.^18^, ^20^, ^53^ Inhomogeneous dose depositions, unavoidable small air gaps, and a quenching effect affecting the Bragg peak region limit the use of films for absolute dose verification and QA procedures.^20^

MC beam models for ion beam therapy are commonly validated using measured in‐depth and lateral profiles.^28^, ^54^, ^55^, ^56^ Reference measurements in water phantoms using single ionization chambers (IC) or their combinations in linear or 3D arrays are considered the standard of practice. However, the extension of these procedures for the validation of beam models interacting with magnetic fields is not straightforward. Dedicated phantoms need to be designed with nonmagnetic materials and the influence on the magnetic field on detector responses needs to be carefully investigated in advance. An alternative validation method of a MC beam model including magnetic fields is proposed through this work using relative EBT3 film dosimetry. This methodology for validation was preferred because of the observed noninfluence of the EBT3 film response in magnetic fields up to 1 T.^46^ Studies on the intrinsic response of detectors using in clinical practice for particle therapy, that is, ionization chambers, in magnetic fields are still missing, to the extent of our knowledge. Moreover, the use of relative dosimetry for MC model validations is extensively used.^30^, ^54^, ^55^, ^57^ Relative comparisons are accurate to adjust the main beam model parameters related to the beam optics and energy distribution. Limitations are rather small, mainly for absolute dose calculations of the MC model, where additional re‐normalization coefficients are required.^56^, ^57^

To test the accuracy of our MC model, IDD and lateral profiles were extracted from the parallel and transverse planar dose distributions in films, mimicking some of the reference measurements using beam data required for treatment planning. Due to the limitations of our experimental setup, range measurements were only possible using parallel film irradiations, while lateral dose distributions were analyzed at different penetration depths for both parallel and transverse configurations. The obtained differences for ΔR_80_ < 0.2 mm, ΔD_mean_ < 1.2%, Δμ_mean_ < 1.0 mm, and ΔFWTM_mean_ < 1.0 mm showed a precision comparable to previous reported data for MC beam model benchmarking.^30^, ^32^, ^57^ Although we consider that this method was sufficient for the purpose of the current work, complementary dosimetric measurements using IC are envisaged using a custom‐designed dedicated water phantom.

## 5 Conclusions

A MC model for proton beams interacting with magnetic fields up to 1 T was developed and benchmarked to experimental data. Beam interactions with custom magnetic field maps were implemented for the first time in GATE, allowing to account not only for homogeneous but also for heterogeneous fringe fields. The very good agreement between measured and calculated proton trajectories in air, as well as in‐depth and lateral dose distributions, demonstrated the accuracy of the model.

The proposed model allows to generate reliable basic input datasets for a research TPS and to support treatment planning and QA procedures for future MRPT systems, reducing considerably the expensive measuring time required to generate the TPS input data.

## Conflict of Interest

The authors have no conflict of interest to report.
